# Carotid Body-Mediated Chemoreflex Drive in The Setting of low and High Output Heart Failure

**DOI:** 10.1038/s41598-017-08142-3

**Published:** 2017-08-14

**Authors:** Rodrigo Del Rio, David C. Andrade, Camilo Toledo, Hugo S. Diaz, Claudia Lucero, Alexis Arce-Alvarez, Noah J. Marcus, Harold D. Schultz

**Affiliations:** 1 Laboratory of Cardiorespiratory Control, Department of Physiology, Pontificia Universidad Católica de Chile, Santiago, Chile; 2 Centro de Investigación Biomédica, Universidad Autónoma de Chile, Santiago, Chile; 3 Department of Physiology and Pharmacology, Des Moines University, Des Moines, IA USA; 4 Department of Cellular and Integrative Physiology, University of Nebraska Medical Center, Omaha, NE USA

## Abstract

Enhanced carotid body (CB) chemoreflex function is strongly related to cardiorespiratory disorders and disease progression in heart failure (HF). The mechanisms underlying CB sensitization during HF are not fully understood, however previous work indicates blood flow *per se* can affect CB function. Then, we hypothesized that the CB-mediated chemoreflex drive will be enhanced only in low output HF but not in high output HF. Myocardial infarcted rats and aorto-caval fistulated rats were used as a low output HF model (MI-CHF) and as a high output HF model (AV-CHF), respectively. Blood flow supply to the CB region was decreased only in MI-CHF rats compared to Sham and AV-CHF rats. MI-CHF rats exhibited a significantly enhanced hypoxic ventilatory response compared to AV-CHF rats. However, apnea/hypopnea incidence was similarly increased in both MI-CHF and AV-CHF rats compared to control. Kruppel-like factor 2 expression, a flow sensitive transcription factor, was reduced in the CBs of MI-CHF rats but not in AV-CHF rats. Our results indicate that in the setting of HF, potentiation of the CB chemoreflex is strongly associated with a reduction in cardiac output and may not be related to other pathophysiological consequences of HF.

## Introduction

Heart failure (HF) is a global public health problem. Currently, it is estimated that 26 million people worldwide are living with this condition, affecting approximately 20% of the world population over 75 years of age, and resulting in more than 1 million hospitalizations annually in both the United States and Europe^1, 2^. HF is a disease with very poor prognosis despite advances in treatment; half of the patients with HF are likely to die in a 4 year time-frame once diagnosed, while more than 50% with severe cardiac dysfunction are likely to die within one year^3^. Development of HF is driven by a complex interaction of multiple pathophysiological stimuli; however chronic hyper-activation of the sympathetic nervous system is widely considered to be a major contributor to disease progression^4^. Importantly, several studies suggest that an enhanced carotid body (CB) chemoreflex contributes to sympathetic activation in HF patients^5^ and experimental models of HF^6^. However, the mechanisms responsible for enhanced CB activity in HF are not fully understood.

The CB is the main peripheral arterial chemoreceptor^7, 8^ mediating a reflex response during acute and chronic alterations in the arterial levels of pO_2_, pCO_2_, pH, glucose, temperature and blood flow^9–11^. In HF patients as well as in animal models, CB function is potentiated during HF leading to sympathoexcitation which in turn further stresses the failing heart. The pivotal contribution of the CB to the progression of HF pathophysiology was shown in recent studies in which CB denervation during the progression of HF in rabbits and rats resulted in normalized autonomic control^12^ and marked improvement in survival^13^. Previous work indicates that decreased antioxidant enzyme expression^14, 15^, increased pro-oxidant enzyme expression^16^, and increased CB angiotensin II levels and angiotensin II type 1 receptor expression^17^ contribute to enhanced CB chemoreflex function in HF. The main pathophysiological signal that triggers these biochemical changes in the CB remains to be elucidated. Ding and colleagues (2011) showed for the first time that control rabbits subjected to chronic carotid artery occlusion using pneumatic cuff occluders resulted in chemoreflex activation and autonomic imbalance that was similar to that observed in HF animals^11^. In addition, Krüppel like factor 2 (KLF2), a mechanosensitive transcription factor that mediates the endothelium response to changes in blood flow and shear stress^18^, has been described to be constitutively expressed in the CB^19, 20^. Haack, *et al*.^20^ showed that in myocardial infarcted HF rats, CB KLF2 expression was significantly lower than in sham animals^20^. This reduction in KLF2 may be mediated by the reduction of blood flow to the CB during low output heart failure (myocardial infarction-CHF [MI-CHF]). Together, these results strongly suggest that potentiation of the CB-mediated chemoreflex function may be related to changes in cardiac output. However, there are no current studies showing the role of cardiac output on CB chemoreflex potentiation nor in cardiorespiratory changes once HF has been developed whereas other factors could play a role in disrupting normal CB function. Therefore, we aimed to determine whether alterations in cardiac output, in the setting of HF, induced CB chemoreflex potentiation and cardiac autonomic imbalance and if these alterations are associated with changes in KLF2 expression.

## Results

**Figure 1:**
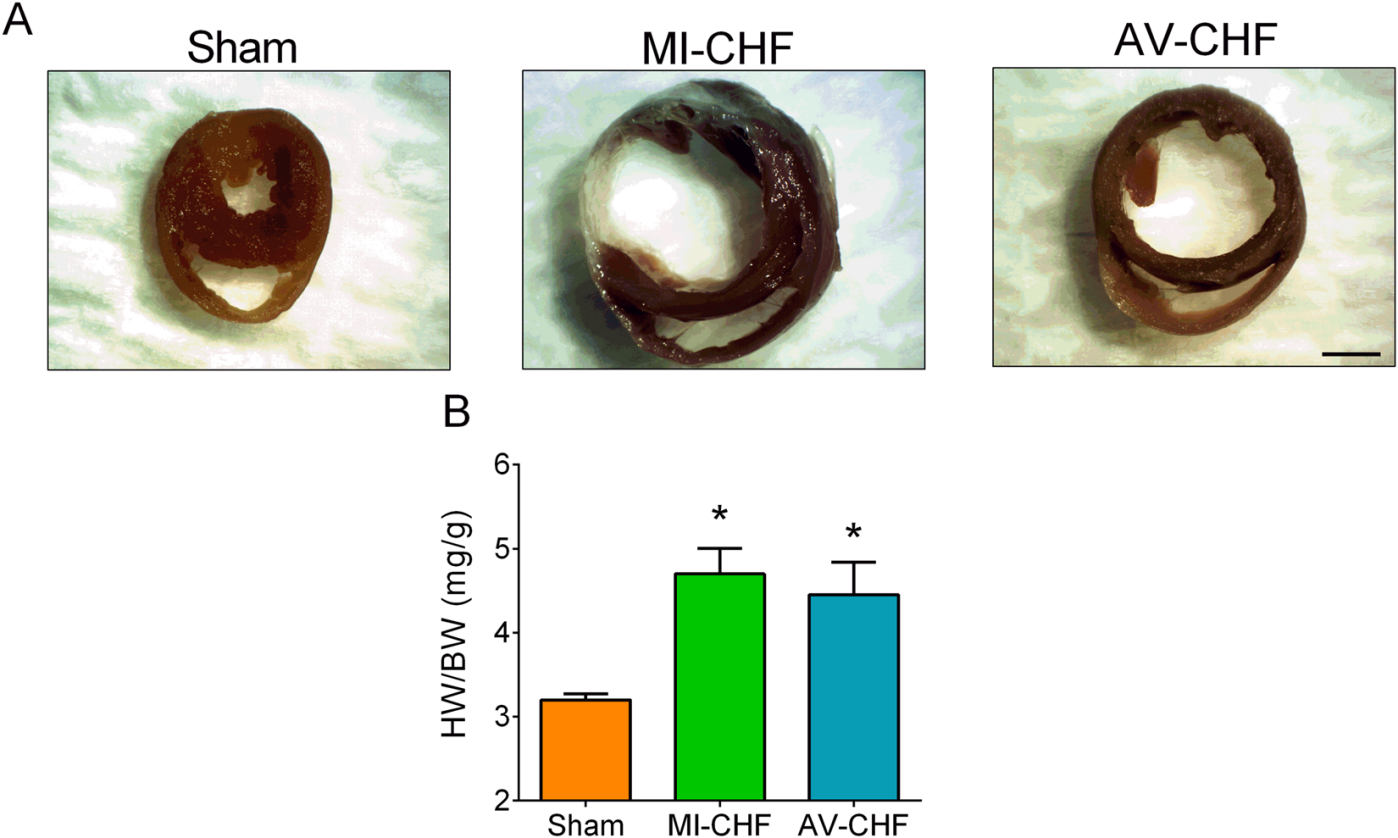
Rats with myocardial infarction (MI-CHF) and arteriovenous fistula (AV-CHF) have similar degrees of cardiac hypertrophy. (**A**) Representative images of hearts obtained in one Sham rat, one rat with MI-CHF and one rat with AV-CHF. Note the large ventricular dilation in both MI-CHF and AV-CHF vs. Sham condition. (**B**) Cardiac hypertrophy (heart weight/body weight [HW/BW]) was evident in both MI-CHF and AV-CHF rats compared to Sham rats. *p < 0.05 vs. Sham condition, Sidak post-hoc test after one-way ANOVA, n = 6 rats.

Echocardiography measurements show that the left ventricular ejection fraction (LVEF) and left ventricular fractional shortening (LVFS) were significantly reduced in MI-CHF rats vs. Sham (LVEF: 44.1 ± 3.7 vs. 77.2 ± 0.8%, n = 10 per group for MI-CHFvs. Sham, respectively) (LVFS: 23.4 ± 2.4 vs. 38.4 ± 0.6%, n = 10 per group for MI-CHF vs. Sham, respectively). In contrast, no differences in LVEF and LVFS between Sham vs. high output CHF (arterio-venous fistula CHF [AV-CHF]) rats were found (see Table 1). Left ventricular end systolic volume (LVESV) and left ventricular end diastolic volume (LVEDV) were significantly increased in MI and AV-CHF rats compared to Sham animals showing clear signs of ventricular dilatation. Accordingly, cardiac hypertrophy was evident in the hearts harvested from both MI-CHF and AV-CHF rats (Fig. 1). Indeed, heart to body weight ratio was significantly increased in MI-CHF and AV-CHF rats (4.7 ± 0.3 and 4.5 ± 0.4 vs. 3.2 ± 0.1 mg/g, n = 6 per group for MI, AV and Sham rats, respectively). No statistical differences were found when comparing MI vs AV-CHF rats.

**Table 1:** Echocardiographic parameters in Sham, MI-CHF and AV-CHF.

	Sham	MI-CHF	AV-CHF
BW (g)	501 ± 28	494 ± 37	545 ± 22
LVESD (mm)	4.3 ± 0.2	8.0 ± 0.3*^,+^	4.8 ± 0.3
LVEDD (mm)	7.3 ± 0.2	9.8 ± 0.3*	8.8 ± 0.2*
LVESV (μl)	86.0 ± 7.5	346.0 ± 25.8*	111.1 ± 14.7*
LVEDV (μl)	278.9 ± 15.0	540.4 ± 30.8*	423.3 ± 24.6*
LVEF (%)	70.5 ± 2.0	35.9 ± 1.4*^,+^	71.0 ± 1.1
LVFS (%)	41.3 ± 1.7	18.2 ± 0.8*^,+^	41.9 ± 0.8

Values are means ± SEM. Body weight (BW), left ventricular end-systolic diameter (LVESD), left ventricular end-diastolic diameter (LVEDD), left ventricular end-systolic volume (LVESV), left ventricular end-diastolic volume (LVEDV), left ventricular ejection fraction (LVEF) and left ventricular fractional shortening (LVFS). *p < 0.05 vs. Sham; and ^+^p < 0.05 vs. AV-CHF, Sidak post-hoc test after one-way ANOVA, n = 10 per group.

### Blood flow and peripheral chemoreflex

**Figure 2:**
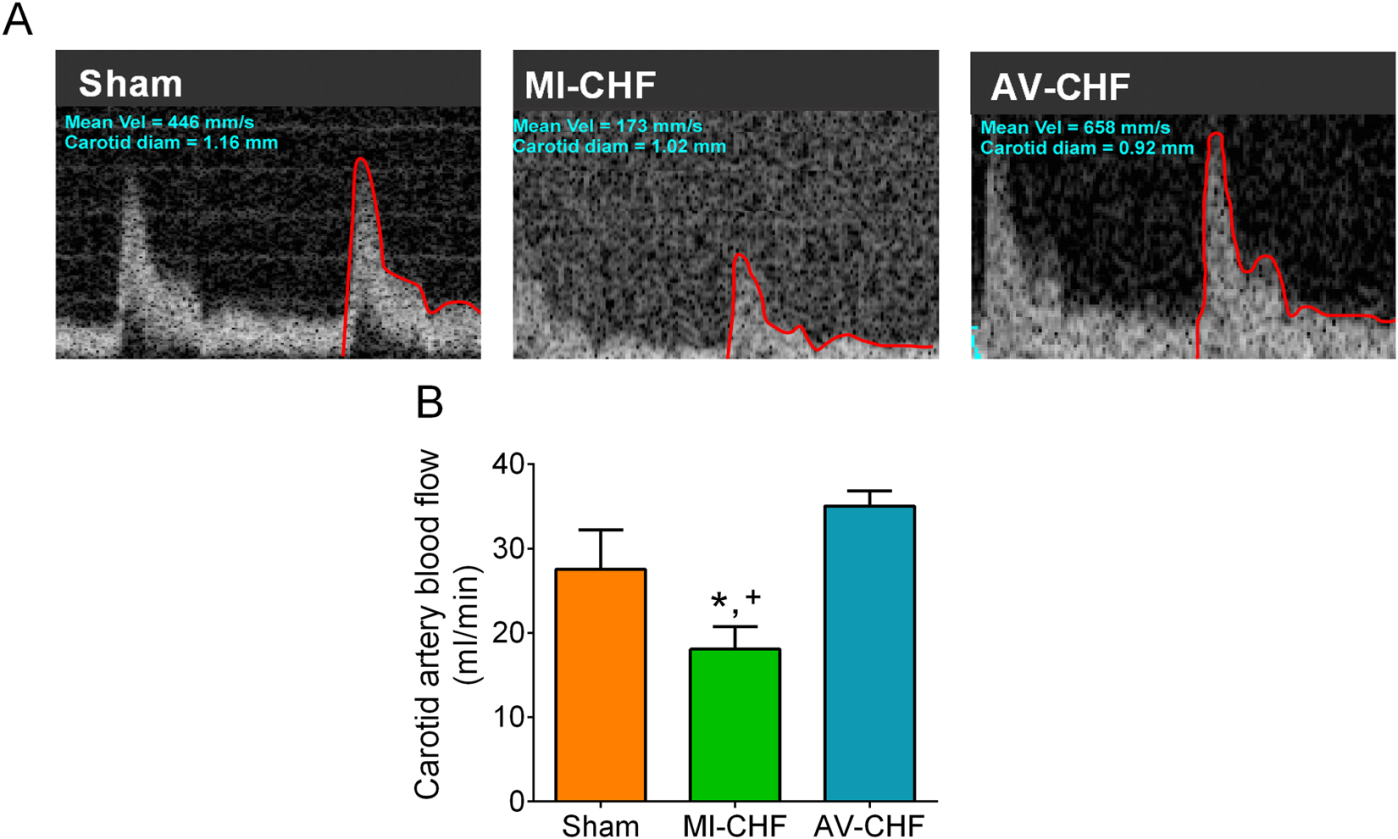
Carotid artery blood flow in low output and high output heart failure. (**A**) Representative traces of carotid artery blood flow, assessed by Doppler at rest in one Sham rat, one MI-CHF rat and one AV-CHF rat. (**B**) Summary data showing carotid artery blood flow. Note that blood flow to the CB region was significantly reduced in MI-CHF vs. Sham. Also note that AV-CHF rats display similar blood flow values compared to Sham rats. *p < 0.05 vs. Sham; ^+^p < 0.05 vs. AV-CHF condition, Sidak post-hoc test after one-way ANOVA, n = 6 rats.

**Figure 3:**
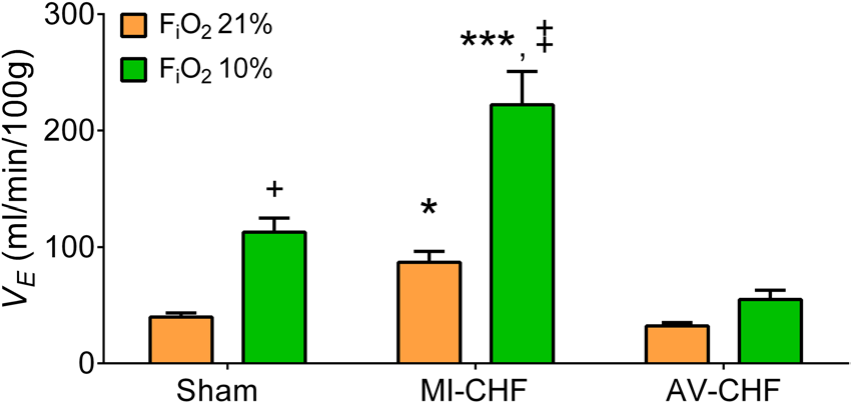
Carotid body-mediated chemoreflex function in heart failure rats. The hypoxic ventilatory response was increased in MI-CHF rats compared to Sham condition. However, in AV-CHF the peripheral chemoreflex ventilatory response to acute hypoxic stimulation was blunted. ***p < 0.001 vs. Sham; *p < 0.05 vs. Sham; ^+^p < 0.001 vs. AV-CHF; and ^+^p < 0.05 vs. AV-CHF, Sidak post-hoc test after one-way ANOVA, n = 6 rats.

Carotid artery blood flow in Sham, MI and AV-CHF rats was used as a surrogate for the estimation of blood perfusion to the CB region (Fig. 2). Carotid artery blood flow was markedly reduced in MI-CHF rats compared to both Sham and AV-CHF Rats (18.1 ± 2.7 vs. 27.5 ± 4.7 and 35.04 ± 1.8 ml/min, n = 10 per group for MI vs. Sham and AV-CHF rats, respectively). In addition, no differences in carotid artery blood flow were found between Sham and AV-CHF group. Consistently with previous findings, CB-mediated chemoreflex drive was significantly increased in MI-CHF rats compared to Sham rats^6^ (Fig. 3). Indeed, minute ventilation (V_E_) in normoxia (F_I_O_2_ 21%) in MI-CHF rats was 87.0 ± 9.4 ml/min/100 g and in Sham rats was 39.9 ± 3.5 ml/min/100 g (n = 6 per group). Interestingly, AV-CHF rats displayed resting normoxic V_E_ values comparable to the ones observed in Sham rats (32.3 ± 2.8 vs. 39.9 ± 3.5 ml/min/100 g, n = 10 per group for AV-CHF vs. Sham, respectively). Hypoxic ventilatory response (F_I_O_2_ 10%) was markedly elevated in MI-CHF rats compared to Sham condition (222.1 ± 28.7 vs. 112.9 ± 12.2 ml/min/100 g of rat, n = 6 per group for MI-CHF vs. Sham, respectively). Interestingly, the ventilatory response to hypoxia in AV-CHF rats was significantly lower compared to the ones obtained in Sham and MI-CHF rats (Fig. 3 n = 6 per group).

### Autonomic imbalance and ventilatory disorders

**Figure 4:**
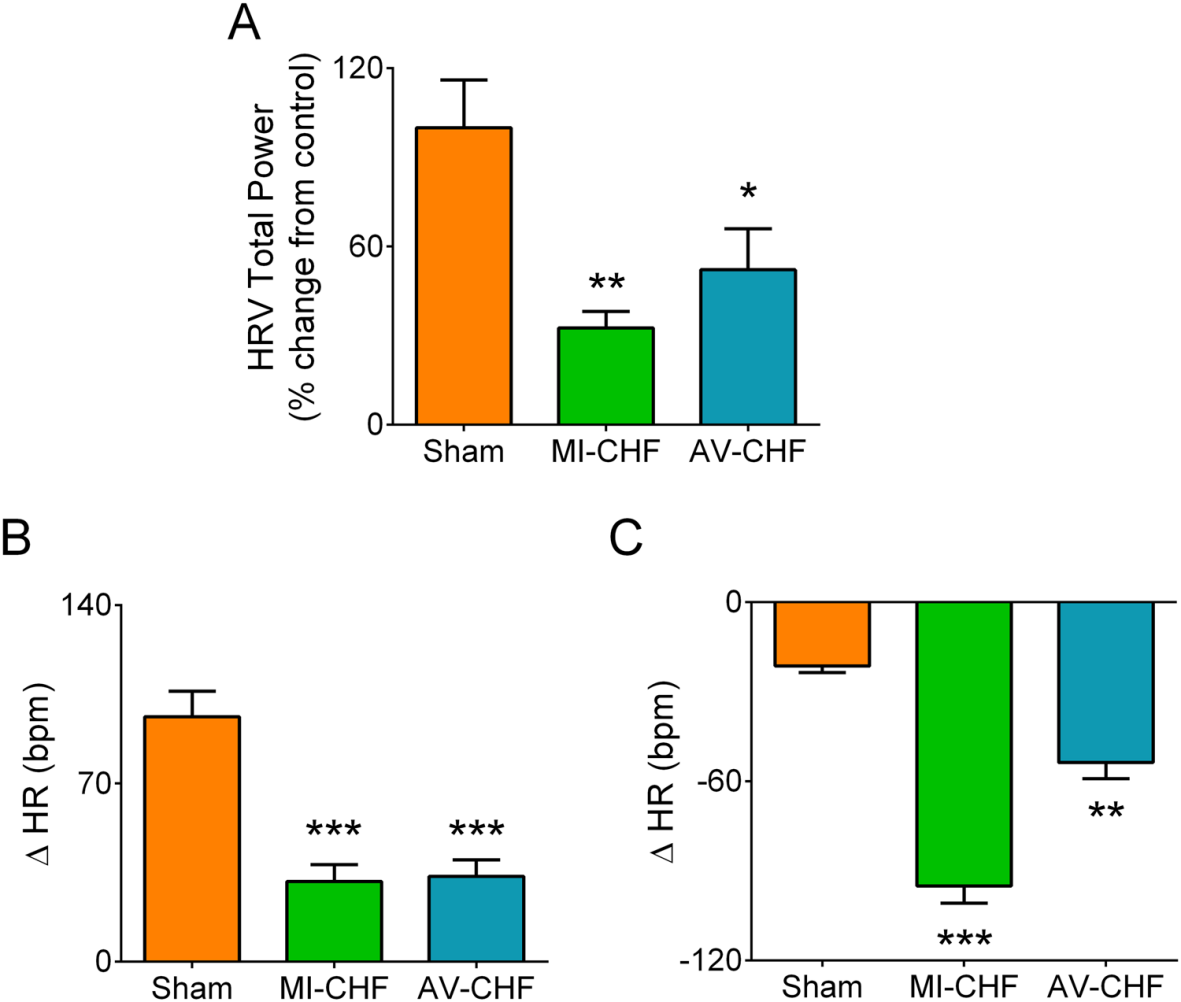
Myocardial infarction (MI-CHF) and arteriovenous fistula (AV-CHF) rats display autonomic imbalance. (**A**) The total power of heart rate variability (HRV) was decreased in MI-CHF and AV-CHF vs. Sham condition. (**B**) MI-CHF and AV-CHF rats display cardiac parasympathetic withdrawal and enhanced sympathetic cardiac tone as evidenced by a decreased tachycardic response (ΔHR) following i.p. bolus of atropine (1 mg/kg) and an increased bradycardic response (**C**) following propranolol (1 mg/kg, i.p.), respectively. ***p < 0.001, **p < 0.01 and *p < 0.05 vs. Sham condition, Sidak post-hoc test after one-away ANOVA, n = 4 rats.

Heart rate variability was decreased in both MI-CHF and AV-CHF rats compared to Sham (Fig. 4A). To fully determine which component of autonomic control to the heart was altered during HF condition, we studied cardiac chronotropic responses during acute sympatho-vagal blockage using propranolol and atropine, respectively. Compared to Sham rats, MI-CHF and AV-CHF rats displayed autonomic imbalance, characterized by an increased cardiac sympathetic outflow and parasympathetic withdrawal (Fig. 4B and C). Indeed, we found that MI-CHF rats showed a 4.5-fold increase in the heart rate (HR) response to sympathetic blockage compared to Sham values (Fig. 4B; n = 4 per group, P < 0.001). Also, AV-CHF rats showed a 2.5-fold increase in the sympathetic control of HR compared to Sham rats (Fig. 4B; n = 4 per group P < 0.01). In addition, we found that MI-CHF rats displayed augmented sympatho-excitation compared to AV-CHF rats despite both were on HF (Fig. 4B). Furthermore, a similar degree of parasympathetic withdrawal was found in MI-CHF and AV-CHF rats compared to Sham (n = 4 per group, P < 0.001). Indeed, the HR change during parasympathetic blockade in MI-CHF rats was 31.6 ± 6.6 bpm compared to 33.5 ± 6.5 bpm obtained in AV-CHF rats and 96.2 ± 9.9 bpm obtained in Sham rats (n = 4 per group) (Fig. 4C). No differences in resting HR or BP were found between groups (Table 2).

**Table 2:** Resting hemodynamics and respiratory physiological parameters in Sham, MI-CHF and AV-CHF.

	Sham	MI-CHF	AV-CHF
SBP (mmHg)	99.2 ± 4.5	96.9 ± 3.8	100.4 ± 5.6
DBP (mmHg)	75.3 ± 2.2	74.7 ± 2.1	69.9 ± 2.4
PP (mmHg)	23.9 ± 8.8	22.3 ± 2.2	30.5 ± 3.7
MAP (mmHg)	83.2 ± 2.5	82.1 ± 2.6	80.1 ± 3.4
HR (bpm)	385.8 ± 7.1	399.5 ± 12.1	361.4 ± 13.9
VT (ml 100 g^−1^)	0.37 ± 0.03	0.47 ± 0.02*^,+^	0.35 ± 0.03
RF (breath min^−1^)	116.4 ± 7.4	168.3 ± 13.3*^,+^	100.1 ± 8.0

Values are means ± SEM. Systolic blood pressure (SBP), diastolic blood pressure (DBP), pulse pressure (PP), mean arterial blood pressure (MAP), heart rate (HR), tidal respiratory volume (VT) and respiratory frequency (RF). *p < 0.05 vs. Sham; and ^+^p < 0.05 vs. AV-CHF, Sidak post-hoc test after one-way ANOVA, n = 6 per group.

**Figure 5:**
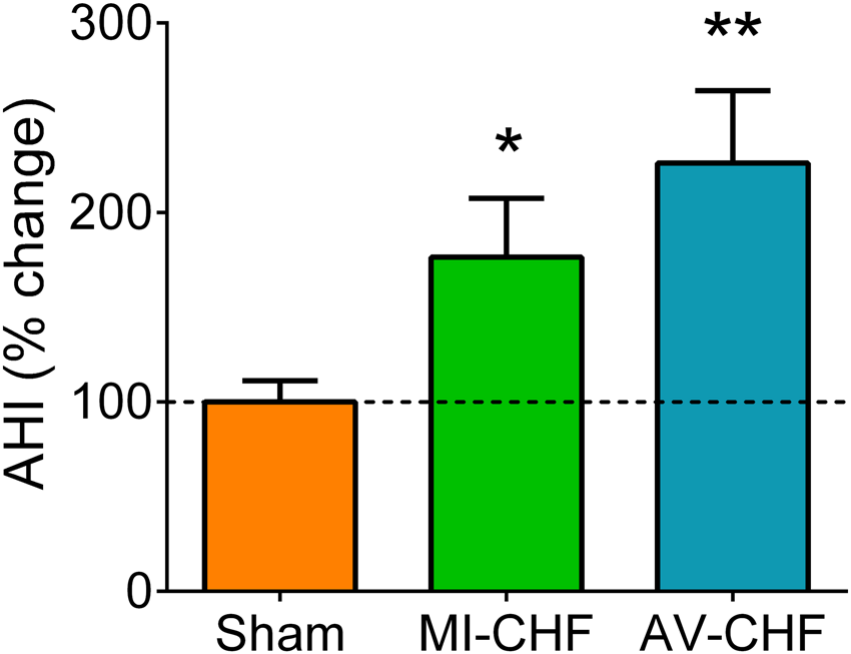
Incidence of apnea-hypopnea in rats with low output heart failure (MI-CHF) and high output heart failure (AV-CHF). The apnea-hypopnea index (AHI) was increased in both MI and AV CHF rats vs. Sham condition. **p < 0.01 and *p < 0.05 vs. Sham condition, Sidak post-hoc test after one-way ANOVA, n = 6 rats.

Resting breathing disturbances were also observed in both MI-CHF and AV-CHF rats when compared to Sham rats. Respiratory disorders were mainly associated with a high prevalence of apneas and hypopneas while rats were resting in normoxia. The incidence of apnea and hypopnea (AHI) were scored in all three groups of rats (Fig. 5). Compared to Sham rats, MI-CHF rats displayed 176 ± 31% more incidence of apneas/hypopneas while AV-CHF rats showed a 226 ± 38% increase in AHI compared to Sham (n = 6 per group).

### Krüppel-like factor 2 (KLF2) expression in MI and AV rats

**Figure 6:**
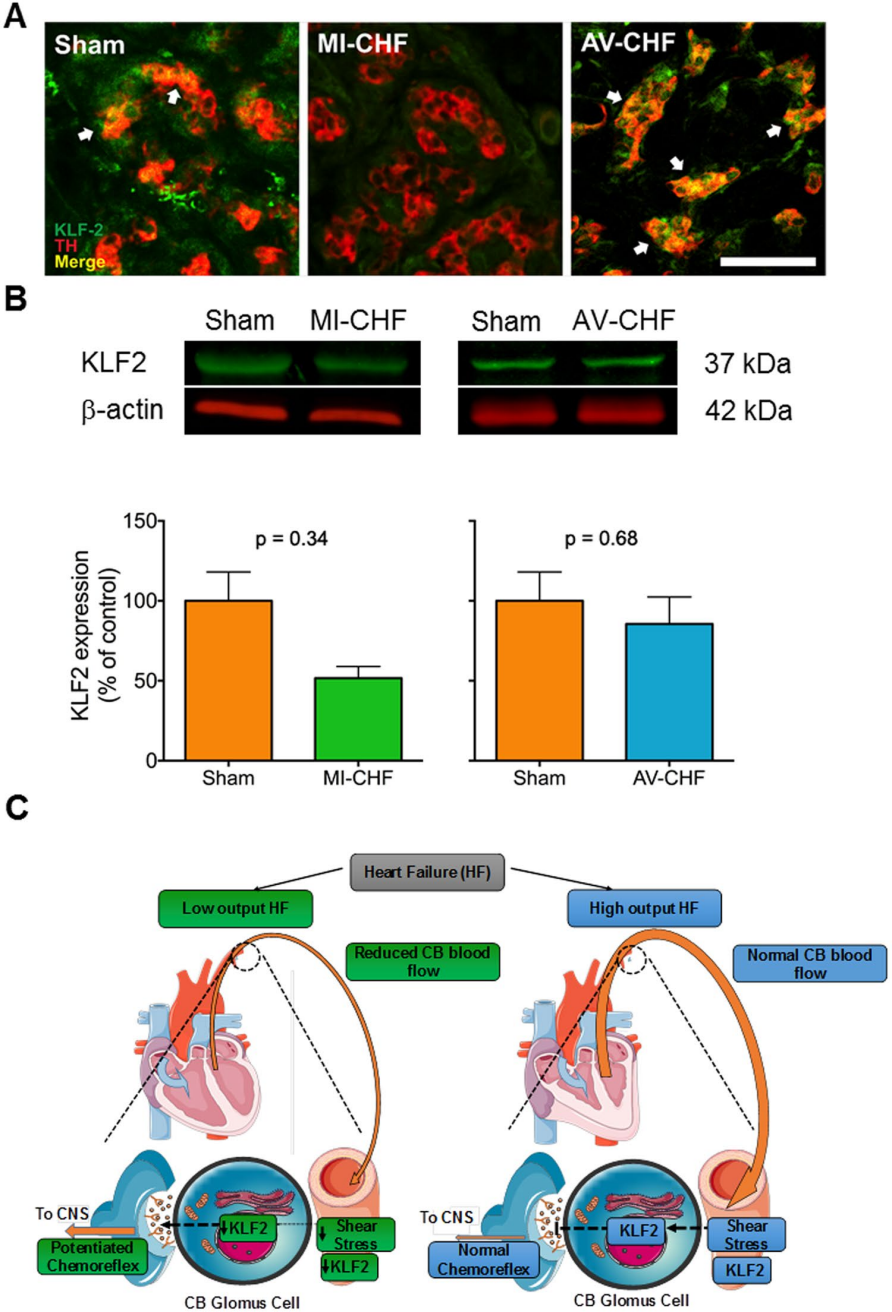
Krüppel-like factor 2 (KLF2) expression is reduced in myocardial infarction (MI-CHF) but normal in arteriovenous fistula (AV-CHF) rats. (**A**) Fluorescence intensity for KLF2 in the CB from MI-CHF was markedly decreased compared to Sham rats. Notably, AV-CHF rats showed similar KLF2 average fluorescence in the CB compared to Sham rats. (**B**) Representative immunoblots for KLF2 obtained in one Sham rat, one MI-CHF rat and one AV-CHF rat and summary data showing the changes in the expression levels of KFL2 in MI-CHF and in AV-CHF (p = 0.34, Sham vs. MI-CHF; p = 0.68, Sham vs. AV-CHF; Wilcoxon sum-rank test, n = 4). (**C**) Proposed model showing the role of blood flow in CB sensitization in low vs high output heart failure. MI-CHF rats display reduced blood flow to the CB that leads to a decreased expression of shear stress-sensitive transcription factors (i.e. KLF2) promoting CB glomus cells hyperactivity and finally ending in an enhanced chemoreflex drive. On the contrary, AV-CHF rats display normal blood flow to the CB with no changes in KLF2 expression and normal CB-mediated chemoreflex drive. Dotted lines: extrapolation of the carotid circulation to the carotid bodies.

KLF2 was constitutively expressed in the CB from Sham rats (Fig. 6). Consistent with previous findings^11, 20, 21^, MI-CHF rats showed a trend for a decreased expression of KLF2 in the CBs compared to Sham rats (p = 0.34; n = 4 per group). In contrast, AV-CHF rats displayed normal KLF2 expression, comparable to that observed in Sham rats (p = 0.68; n = 4 per group).

## Discussion

In the present study we show that CB chemoreflex potentiation is strongly associated with reduced cardiac output in the setting of HF. The main findings of the present study are: i) low output HF rats show a trend to display reduced KLF2 expression levels in the CB and an enhanced CB chemoreflex drive, ii) high output HF rats have normal KLF2 expression in the CB and reduced CB chemoreflex function, and iii) autonomic imbalance and breathing disturbances in high output HF are not related to CB chemoreceptor potentiation.

Previous studies indicate that CB chemoreflex function is enhanced during the development of low output HF^5, 6^. Furthermore, the presence of heightened CB chemoreflex drive has been associated with the activation of the sympathetic nervous system and with the development of altered resting breathing patterns^6, 20, 22, 23^. Indeed, HF patients with a reduced EF as well as animal models exhibit increased chemosensitivity^5, 15, 19^. Accordingly, we have previously shown in two low output HF models that irregular breathing patterns are critically dependent on the CB inputs since chronic denervation of the CB results in normalization of breathing^13^. In the present study, we confirm and extend previous findings showing the relevance of the CB to altered breathing patterns on low output HF. We show for the first time that in experimental high output HF the CB chemoreflex function remains normal, comparable to that observed in control animals. Interestingly, AV-CHF rats displayed similar incidence of apneas and hypopneas compared to MI-CHF rats despite the marked difference in CB chemoreflex drive. It is worth noting that human HF patients with no reductions in EF also displayed signs of periodic breathing^23^. Therefore, we hypothesize that other factors may play a role in the development of disordered breathing in AV-rats. One potential mechanism that may contribute to cardiorespiratory alterations in AV-CHF rats may be related to the activation of brainstem central nervous system pathways associated with cardiovascular and breathing regulation. Indeed, Shigematsu *et al*.^38^ showed that AV-CHF rats displayed neural activation at the nucleus of the solitary tract, a well know cite for the integration of respiratory and baroreflex activity. Future studies will be needed to understand the precise mechanisms that underlie ventilatory disturbances in high output HF.

Excessive sympathetic outflow is a hallmark of HF, and contributes to cardiac arrhythmias, cardio-renal syndrome, and deterioration of cardiac function^22–26^. It is well established that acute and/or chronic activation of the CBs enhances sympathetic drive^11, 14, 15, 22, 23, 27^. Our previous studies indicate that heightened CB chemoreflex drive contributes to cardiac autonomic imbalance in HF^12, 13^. In the present study we showed that rats with MI induced HF exhibit enhanced CB chemoreflex drive and cardiac autonomic imbalance characterized by sympathoexcitation and parasympathetic withdrawal. In addition, we show that rats with AV induced HF also displayed autonomic imbalance. However, in this model of HF, the autonomic change did not appear to be related to changes in the CB chemoreflex since no heightened CB chemoreflex drive was observed.

It has been shown that patients with HF with normal EF show an increased cardiac sympathetic drive^28–30^. It is well known that angiotensin II acting mainly through the activation of the angiotensin type 1 receptor, located in cardiovascular control neurons in the central nervous system induces sympathoexcitation^31–34^. Furthermore, it has been shown that angiotensin peptides mediate chronic increases in sympathetic outflow in several cardiovascular diseases including HF^34–37^. Shigematsu *et al*. showed that AV-rats have increased expression of the angiontensin converting enyme in the brainstem suggesting increased levels of angiotensin II in the central nervous system, particularly in areas related to cardiorespiratory control^38^. Also, angiotensin II-mediated increases in renal sympathetic nerve activity have been found to be increased by 3-fold in AV-CHF rats compared with control animals^38^. Together, these data suggest that increases in central and/or systemic levels of angiotensin II during the progression of AV-CHF may contribute to cardiac autonomic imbalance. Further studies should focus on the mechanism underlying neuronal activation in key central cardiovascular nuclei (i.e. paraventricular nuclei, rostral ventrolateral medulla, nucleus of the solitary tract).

It has been previously shown that reductions in blood flow to the CB region in healthy animals are sufficient to cause peripheral chemoreflex sensitization^11, 21^. In these studies, chronic reductions in CB blood flow in rabbits with otherwise normal cardiac function resulted in chemoreflex potentiation and alterations in angiotensin and nitric oxide metabolism that mimics that observed in HF rabbits with reduced EF. In the present study, we showed that in the setting of HF the potentiation of the CB-mediated chemoreflex drive is extremely dependent on the reductions in EF since only MI-CHF rats, a low output HF model, and not AV-CHF rats, a high output HF model, showed a significant increase in CB chemoreflex sensitivity.

Furthermore, our data suggest that changes in cardiac output may trigger KLF2 expression changes in the CB. KLF2 is a master transcription factor involved in the vascular responses to shear stress at least in part by regulating the expression of angiotensin converting enzyme (ACE) and nitric oxide synthase (NOS)^18, 21^. Importantly, previous studies from our lab showed that an increase in angiotensin II and, a reduction in NO availability in the CB are both involved in chemoreflex potentiation in rabbits and rats with HF with reduced EF^16, 19, 39^. Accordingly, in the present study we found a reduced CB KLF2 expression in rats that displayed a marked reduction in carotid artery blood flow (MI-CHF rats). Also, MI-CHF rats showed a significant increase in CB-mediated chemoreflex drive. In contrast, AV-CHF rats with normal carotid artery blood flow had normal KLF2 expression and no CB chemoreflex potentiation. Together, these results suggest that KLF2 expression changes within the CB are associated with the changes in ejection fraction in HF (Fig. 6).

Several limitations are inherent from our study. While CB chemoreflex potentiation appears to be related to decreases in ejection fraction in HF, we cannot rule out the possibility that altered cerebral blood flow during low output HF also contribute to enhanced the reflex response rising from the CB inputs. Indeed, It has been proposed that reductions in cerebral blood flow could reset chemoreflex gain^41–43^. However, the compensatory adjustments following brain hypo-perfusion results in brain blood vessels dilation which in turn helps to wash out the build-up in CO_2_. Therefore, decreases in cerebral blood perfusion to chemosensitive related areas should result in decreases and not in increases in chemoreflex sensitivity. Unfortunately, there are no studies showing the contribution of cerebral blood flow changes on chemoreflex sensitiviy in HF. Future studies are needed to evaluate the effects of decreased blood flow to the brain on CB-mediated chemoreflex responses in the setting of HF.

In summary, low output HF rats present reduced CB KLF2 expression, enhanced chemoreflex drive, breathing instability and increased apnea incidence, and cardiac autonomic dysfunction compared to Sham rats. In contrast, high output HF rats showed normal CB KLF2 expression and CB chemoreflex function. Remarkably, high output HF rats showed comparable levels of breathing disturbances and cardiac autonomic imbalance compared to the ones observed in low output HF rats despite the presence of a reduced peripheral chemoreflex drive. Another factors may account for the elevated sympathetic activity and breathing irregularity in high output HF. Together, our results suggest that autonomic imbalance and ventilatory disorders in high output HF are not dependent on the enhancement of the peripheral chemoreflex drive. Future studies should focus on identifying the mechanisms that subside the development of autonomic and ventilatory impairment in high output HF.

## Methods

Thirty male Sprague-Dawley rats, weighing between 360 and 430 g, were used in these experiments. All experiments were approved by the Bioethical Committee of the Universidad Autónoma de Chile and the Institutional Animal Care and Use Committee of the University of Nebraska Medical Center, and were carried out under the guidelines of the American Physiological Society, the Guía para el Cuidado y Uso de los Animales de Laboratorio from CONICYT and the National Institutes of Health Guide for the Care and Use of Laboratory Animals.

### Rat model of low-output chronic heart failure

Low output HF (MI-CHF) was produced by coronary artery ligation induced myocardial infarction (MI-rats) as previously described. Briefly, rats were anesthetized (2% isoflurane/98%O_2_) and mechanically ventilated then a left thoracotomy was performed. The left anterior descending coronary artery was ligated near its branch point from the aorta with a 6–0 silk suture. Following these maneuvers, the thorax was closed and the air within the thorax was evacuated. All animals were allowed to resume spontaneous respiration and recover from anesthesia. Rats were then housed in a temperature and humidity control environment with ad libitum access to food and water. All experiments were performed at 8 wk. after coronary artery ligation.

### Rat model of high-output chronic heart failure

High-output HF (AV-CHF) was produced by volume overload induced by arterio-venous fistula (AV-rats) using the needle technique as described previously^40^. Briefly, rats were anesthetized (2% isoflurane/98%O_2_) and a midline abdominal incision was made to fully expose the aorta and vena cava. Then a longitudinal incision was made in the inferior vena cava and a fistula was created between the two vessels using 1.20 × 40 mm needle (BD PrecisionGlide). The opening in the vena cava was then closed with tissue adhesive (Hystoacryl, Braun). Then, the abdomen was closed in layers. Rats were then housed in a temperature and humidity control environment with ad libitum access to food and water. All experiments were performed at 8 wk.

### Sham animal surgeries

MI-Sham rats (n = 6) and AV-Sham rats (n = 4) were grouped into one single control groups. Sham operated rats were prepared in the same manner (described previously) but did not underwent MI and/or AV, respectively. All experiments were performed at 8 weeks post surgery.

### Cardiac echocardiography and carotid artery blood flow doppler

Cardiac function and the degree of HF were determined by echocardiography (Vevo 770; Visualsonics, Inc.). Under isoflurane anesthesia, a two-dimensional, short-axis view of the left ventricle (LV) was obtained at the level of the papillary muscles. M-mode tracings were recorded through the anterior and posterior LV walls, and anterior and posterior wall thicknesses (end-diastolic and end-systolic) and LV internal dimensions were also measured. Rats with ejection fraction (EF) of less than 45%, were considered to be in low output HF while rats showing preserved EF but with clear signs of chamber dilation (2.5 standard deviations from the mean obtained in control rats) where considered to be in high output HF. During the same session, rats underwent carotid artery Doppler to estimate blood flow.

### Radiotelemetry monitoring of arterial blood pressure and heart rate

After 4 weeks of either MI or AV surgery, rats were implanted with a radio-telemetry device (Physiotel TA11PA-C40, Data Science International, USA) for measurement of blood pressure (BP) under 2% isoflurane anesthesia. Briefly, the tip of a pressure-sensing catheter was inserted into the left femoral artery and advanced to place the tip in the aorta. After 14 days of recovery, continuous changes in BP and heart rate were measured in conscious freely moving animals at rest.

### Evaluation of cardiac autonomic balance

The total power of heart rate variability (HRV) was calculated as an indirect measure of autonomic control to the heart as previously described^11^. Briefly, BP recordings were obtained at 2 kHz over a 60 min period during the resting breathing test. Heart rate (HR) was derived from the inter-pulse interval. HRV was analyzed using the HRV extension for LabChart 7 software (AD Instruments) over a 10-min recording characterized by the absence of movement-induced artifacts. Power spectral analysis was performed to calculate the total power of the HRV by integration of the whole spectrum. In addition, to estimate sympatho-vagal control of heart rhythm, the chronotropic HR responses during acute sympathetic blockage with propranolol (100 mg/kg i.p.) and parasympathetic blockade with atropine (100 mg/kg i.p.) was study.

### Ventilatory CB-mediated chemoreflex function and apnea/hypopnea incidence

Tidal volume (V_t_), respiratory frequency (RF), and minute ventilation (V_*E*_: V_t_ x RF) were determined by unrestrained whole body plethysmography. Rats where placed in a Plexiglas chamber. The chamber was sealed, except for an inlet and outlet port that allowed a continuous flow of air through the chamber. Animals were kept for at least 1 hour before chemoreflex measurements were taken. The plethysmograph was set-up in a dedicated room in the animal facility with controlled temperature and humidity. Tidal volume was measured by temporarily (15–30 s) sealing the air ports and measuring the pressure changes in the sealed chamber using a Validyne (MP-45) differential pressure transducer connected to an amplifier and a PowerLab System (AD Instruments). Calibration was performed by injecting known volumes of air to the chamber once the experiment was finished. Calibration was performed each day experiments were conducted. Resting breathing was recorded for 2 h while the rats breathed room air. Apnea episodes (cessation of breathing ≥ 3 breaths) and hypopneas (reductions ≥ 50% in V_t_) were average during resting breathing recordings. The CB-mediated chemoreflex function was estimated by allowing the rats to breathe hypoxic (10% O_2_/balance N_2_) gas for 2–5 min under isocapnic conditions as previously described^6^. All recordings were made at an ambient temperature of 25 ± 2 °C. During chemoreflex testing, respiratory variables (RF and V_t_) were averaged for at least 20 consecutive breaths over a period of 4 min of inspired hypoxic challenges.

### Krüppel-like factor 2 expression

KLF-2 protein expression within the CB was assessed using immunostaining as previously described^20^. Briefly, rats were anesthetized and perfused intracardially with buffered paraformaldehyde (PFA 4%, Sigma) for 10 min. The carotid artery bifurcations including the CBs were harvested from the rats and postfixed by in the same fixative solution for 12 h at 4 °C. Tissue was then cryopreserved using a sucrose gradient (5%, 10%, 20% in PBS), and embedded in OCT. Sections containing the CB (10 μm) were blocked/permeabilized in 0.5% Triton X-100, 2% fish skin gelatin (Sigma-Aldrich), 1%BSA in PBS for 1 hour at RT. Sections were incubated overnight at RT with a mixture of an goat anti-KLF2 polyclonal antibody (1:100 in the same blocking media, Abcam) and an mouse anti-TH monoclonal antibody (1:250 in the same blocking media, Millipore) the latter used as a positive control for CB chemoreceptor (glomus) cell recognition. After being washed with PBS, tissue sections were incubated for 1 h with a mixture of Alexa-Fluor 488 rabbit anti goat IgG (1:200, Molecular Probes) and Alexa-Fluor 546 rabbit anti-mouse IgG (1:200, Molecular Probes). Finally, sections were mounted (Vectashield, Vector Laboratory) and visualized using a confocal laser microscope (Leica).

KLF2 protein expression was studied as previously described^20^. Briefly, after euthanizing the rats, the carotid sinus region containing the CBs, was quickly removed and snap frozen on dry ice and stored at −80 °C. Tissue was lysed in 200 µL of RIPA buffer with fresh protease inhibitor cocktail (Sigma Aldrich, St. Louis, MO) and total protein concentration was assessed using a Thermo Scientific BCA Assay kit (Waltham, MA) prior to Western blot analysis. Samples were loaded onto a 7.5% SDS-PAGE gel (50 µg/20 µl per well) and electrophoresis was performed. Fractionated proteins were then transferred to a PVDF membrane (Millipore, Billerica, MA) and the membrane probed with the following antibodies overnight: rabbit anti- KLF2 (1:750, Novus Biological), and/or mouse anti- β-actin (1:500–1:1,000, Santa Cruz). Following washes with PBST, the appropriate secondary antibodies (Li-Cor Biosciences, Lincoln, NE) were added to each membrane. Blots were developed using a Li-Cor Odyssey scanner and quantitative analysis of band densitometry was performed using the Li-Cor Odyssey software. The relative amount of protein of interest was calculated as the ratio of intensity of the band relative to the intensity of the housekeeping.

### Statistical Analysis

Data were expressed as means ± S.E.M. All data were subjected to Shapiro-Wilk normality test. The differences on hypoxic ventilatory response was analyzed by two-ways ANOVA (2 x 3), following Sidak post-hoc. To determine the differences between groups on echocardiography parameters, resting hemodynamics and respiratory physiological parameters, cardiac hypertrophy, carotid artery blood flow, autonomic control and AHI, One-way ANOVA followed by Sidak post-hoc was used. For KLF-2 expression (nonparametric data), the Wilcoxon sum-rank test was employed. A p value of <0.05 was considered statistically significant.
